# The Structure Design of Piezoelectric Poly(vinylidene Fluoride) (PVDF) Polymer-Based Sensor Patch for the Respiration Monitoring under Dynamic Walking Conditions

**DOI:** 10.3390/s150818801

**Published:** 2015-07-31

**Authors:** Kin-Fong Lei, Yi-Zheng Hsieh, Yi-Yuan Chiu, Min-Hsien Wu

**Affiliations:** 1 Graduate Institute of Medical Mechatronics, Chang Gung University, Taoyuan 33302, Taiwan; E-Mail: kflei@mail.cgu.edu.tw; 2 Department of Mechanical Engineering, Chang Gung University, Taoyuan 33302, Taiwan; 3 Graduate Institute of Biochemical and Biomedical Engineering, Chang Gung University, Taoyuan 33302, Taiwan; E-Mails: ed9588382@hotmail.com (Y.-Z.H.); m9607216@mail.ntust.edu.tw (Y.-Y.C.)

**Keywords:** respirations, poly(vinylidene fluoride) (PVDF), sensor patch, dynamic condition

## Abstract

This study reports a piezoelectric poly(vinylidene fluoride) (PVDF) polymer-based sensor patch for respiration detections in dynamic walking condition. The working mechanism of respiration signal generation is based on the periodical deformations on a human chest wall during the respiratory movements, which in turn mechanically stretch the piezoelectric PVDF film to generate the corresponding electrical signals. In this study, the PVDF sensing film was completely encapsulated within the sensor patch forming a mass-spring-damper mechanical system to prevent the noises generated in a dynamic condition. To verify the design of sensor patch to prevent dynamic noises, experimental investigations were carried out. Results demonstrated the respiration signals generated and the respiratory rates measured by the proposed sensor patch were in line with the same measurements based on a commercial respiratory effort transducer both in a static (e.g., sitting) or dynamic (e.g., walking) condition. As a whole, this study has developed a PVDF-based sensor patch which is capable of monitoring respirations in a dynamic walking condition with high fidelity. Other distinctive features include its small size, light weight, ease of use, low cost, and portability. All these make it a promising sensing device to monitor respirations particularly in home care units.

## 1. Introduction

The measurement of important physiological parameters (e.g., respiratory rate, heart rate, body temperature, or blood pressure) is widely utilized to evaluate the basic functions of human body in general health care units. Among the vital signs, the measurement of respiratory rate can provide us with the information about the status of diseases or the events of clinical emergency (e.g., sudden infant death syndrome (SIDS) [1], asphyxia [2], or obstructive sleep apnea (OSA) [3,4]). For the people with high risk of the above symptoms, it is, therefore, of great importance to monitor their respiratory rate in a routine or real-time manner.

The commercially-available vital sign monitors commonly used in the clinical setting can achieve multiple physiological parameter monitoring in a real-time and high-precision manner. In general, however, they are costly and bulky, and thus, could hamper their practical utilizations for patient home care. In order to assist the patients to monitor their respiratory rate during home care, a simple and portable sensing device is particularly required. To address the practical needs, sensing devices with different working mechanisms for respiration monitoring have been extensively proposed [5]. For example, the sensing devices based on the detections of nasal/oral airflow [6], or thorax movement [6] can achieve real-time monitoring of respiratory rate. For the nasal/oral airflow-based sensing devices, airflow sensors are located in front of nasal/oral region to detect the air flow generated during respirations. For the latter, the electrodes are attached on a chest wall to detect the changes of transthoracic impedance during the respiratory movements. Although some of these devices for respiration monitoring have been utilized clinically there are some technical disadvantages that could restrict their practical applications particularly in patient home care. Firstly, some of these devices might require skilled health personnel to setup and to operate so as to acquire reliable data. In addition, these devices are, to some extent, not comfortable and convenient to the end users, particularly for long-term respiration monitoring (e.g., the use of air collector or face mask in the airflow sensors [7]). During dynamic conditions (e.g., walking), more importantly, these devices might not be able to detect respiratory signals due to the issue of dynamic noises.

To achieve respiration monitoring, the use of piezoelectric poly(vinylidene fluoride) (PVDF)-based sensing mechanism holds great promise due to its unique material properties [5,8,9,10,11,12,13]. PVDF polymer, a kind of piezoelectric material, can generate electrical signals while it is mechanically stimulated [14]. In addition, it is a thin, flexible, light-weight, and inexpensive polymer. These advantageous features make it an ideal material of choice for the fabrication of portable or disposable sensing devices for respiration monitoring [14,15,16]. The PVDF polymer-based devices for physiological signal detections have been actively proposed. For the study of sleep quality, for example, PVDF cable sensors, or PVDF sensor array-integrated mattress were developed, by which the cardiac and respiratory actions can be recorded in a lying posture [5,8]. Moreover, a PVDF-based wearable belt-type sensor was proposed, in which the belt was tightly fasten around a chest wall to detect the respiration-driven movements [9,10]. Although abovementioned devices, overall, have been demonstrated feasible for the monitoring of respirations they are complicated either in fabrication or application. Therefore, these devices are more suitable for the use in the medical centers rather than home care units. To address the issue, more recently, a simple, small-size, light-weight, and user-friendly PVDF polymer-based sensor patch for respiration detections was proposed [17]. In operation, the patch-type sensor is simply adhered to a human chest wall. The working mechanism is based on the periodical deformations on a chest wall during the physiological movements of respirations, which in turn mechanically stimulate the piezoelectric PVDF film to generate the corresponding electrical signals. In that work, a freely-movable curved structure of PVDF film was designed to enhance the detection signals [17]. Although the sensing device was proved feasible to detect respirations with high precision, again, it might not be capable of detecting respirations during dynamic conditions (e.g., walking) due to dynamic noise problem.

In order to develop a sensing device for home-care respiratory monitoring, a sensing device capable of detecting respirations during daily dynamic conditions (e.g., walking) will be required. To address the practical requirement, a patch-type PVDF-based sensor capable of detecting respirations under dynamic walking condition was proposed in this study. Different from the similar sensor patch reported previously [17], the proposed sensor patch structurally consists of three layers, in which two elastic polydimethylsiloxane (PDMS) polymer-based cover layers were used to completely encapsulate a thin PVDF sensing film. Different from the previously-reported sensor patch, the working mechanism of respiration signal generation is based on the periodical deformations on a human chest wall during the respiratory movements, which in turn mechanically stretch the piezoelectric PVDF film to generate the corresponding electrical signals. Based on the structure of sensor patch abovementioned, the sensor patch can be regarded as a mass-spring-damper mechanical system. Therefore, it is capable of more significantly reducing the signal noises generated in a dynamic condition in comparison with an undamped system (e.g., the previously-reported sensor patch [17]). Experimental results revealed that the respiration signals generated by the proposed sensor patch were in concordance with that based on a commercial respiratory effort transducer (RET) both in static (sitting) and dynamic (walking) conditions. In terms of the measurement of respiratory rates, moreover, results also exhibited that there was no statistic difference (*p* > 0.05) between the respiratory rates measured by the proposed sensor patch and by a commercial RET both in the static and dynamic conditions explored. All abovementioned evaluations could suggest that the proposed sensor patch was able to faithfully respond to the physiological movements of respirations under the conditions explored. As a whole, the simple, small-size, and light-weight sensor patch capable of detecting respiration signals both in static and dynamic conditions is found particularly useful for real-time respiration monitoring in patient home care.

## 2. Materials and Methods

### 2.1. Sensor Design and Experimental Setup

**Figure 1:**
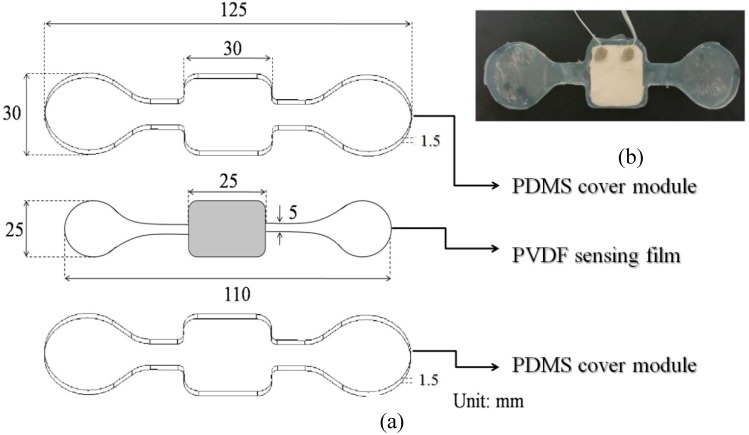
(**a**) Schematic illustrations of the structure of sensor patch, and (**b**) the photograph of sensor patch (**upper right**).

As illustrated in Figure 1a, the proposed sensor patch is structurally composed of three layers, including a top and bottom polydimethylsiloxane (PDMS) polymer-based cover layer [Thickness (T): 1.5 mm] (Sylgard^®^ 184, Dow Corning, Midland, MI, USA), a piezoelectric PVDF [poly(vinylidene fluoride)] polymer sensing film layer (T: 25 μm) (Pro-Wave Electronic Corp., New Taipei City, Taiwan). Briefly, PDMS is an elastic transparent, gas-permeable and biocompatible material that is widely used in biomedical field. In this study, the two PDMS covers were not only designed to create the main structure of the proposed sensor patch but also to completely encapsulate the thin PVDF sensing film sandwiched in-between. The two PDMS covers were fabricated through a replica molding process. Briefly, two polymethylmethacrylate (PMMA) molds with the desired structures were created using a CNC miller (EGX-400, Roland Inc., Hamamatsu, Japan) with a 0.4 mm drill bit (rotational speed: 26,000 rpm). In the following replica molding process, PDMS was prepared by thoroughly mixing the PDMS pre-polymer with a curing agent in a ratio of 10:1 by weight according to the manufacturer’s instructions. The polymer was then deaerated under vacuum to remove any air bubbles generated during mixing. Then the mixture was poured onto the two fabricated PMMA molds and cured at 70 °C for 1 h. The cured PDMS covers were then obtained after a de-molding process. For the PVDF film, the central rectangular area is defined as the sensing area, outside this region, the PVDF polymer on the film surface was removed physically (Figure 1a). Moreover, two printed electrodes (each area: 8 × 8 mm; thickness: 150 µm; material: silver paste) on the surfaces of the PVDF sensing film were designed for signals transduction and ground connection, respectively. Both of them were connected to shield cables to prevent electrical noise. The photograph of the assembled sensor patch is shown in Figure 1b.

In order to describe a system and to predict its response, systems can be mathematically modeled as differential equations. A mass-spring-damper model, a second order model, is typically used to describe various systems in the real world, such as vehicle suspension and RLC (resistor, inductor, and capacitor) circuit. Based on the change of the parameters of mass, spring, and damper, the system response can be adjusted and controlled. Typical descriptions of the system response are rise time (the time taken by a vibration amplitude to change from 10% to 90%), overshoot (the maximum amplitude that exceeding the steady-state amplitude), and settling time (the time taken the system transients to decay). In this work, we described the proposed sensor patch using the mass-spring-damper model. The flexibility of the PDMS material was modeled as the parameter of spring and could not be adjusted because it is the material property. The thickness of the encapsulating PDMS cover layer was modeled as the parameter of damper.

**Figure 2:**
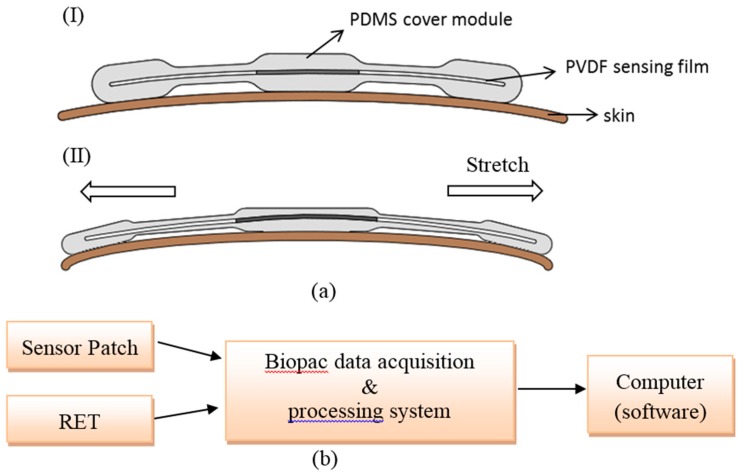
(**a**) Schematic illustration of the working mechanism of sensor patch: (I) the rest state; (II) the mechanical stretch of sensor patch due to the deformations on the chest wall of human body during the physiological movements of respirations; (**b**) the illustration of overall experimental setup.

In this study, the working mechanism of respiration signal generation is based on the periodical deformations on the chest wall of human body during the physiological movements of respirations, which in turn mechanically stretch the piezoelectric PVDF film encapsulated in PDMS material to generate the corresponding electrical signals. The generated analog signals were inputted into a data acquisition and processing system (BIOPAC Systems, Inc., Goleta, CA, USA) through two wires that connected the sensor patch and the system. The system acted to process the signals and to convert the analog signals to the digital signals (sampling rate: 1000 Hz). The digital signals were then acquired and processed through a computer. The signals were then analyzed numerically by Matlab^®^ software (The MathWorks, Inc., Natick, MA, USA). The overall experimental setup is illustrated in Figure 2b.

### 2.2. Signal Processing and Sensing Performance Evaluation

In this study, the digital signals collected through (1) the proposed sensor patch; (2) commercial respiratory effort transducer (RET) were then analyzed using the Matlab^®^ software. To quantitatively determine the respiratory rate, a band pass filter was adopted. For the acquisition of respiratory signals, briefly, the pass band of the filter was set at 0.1 Hz–0.5 Hz, which is capable of covering physiological respiratory rate [18]. In this study, furthermore, the power spectral density (PSD) spectrum based on the Welch *et al.* (1967) [19] was used to determine the average respiratory rate.

To verify the design of the proposed sensor patch to prevent dynamic noises, experimental investigation was carried out. Briefly, the respiration detections based on the use of the previously-reported sensor patch [17] and the proposed sensor patch were performed. The respiration detections were carried out in static (sitting) and dynamic (walking) conditions, and were compared with that based on a commercial RET under the corresponding conditions. In operations, the proposed sensor patch (Figure 1b) and the previously-reported sensor patch were adhered to the chest wall of the same human body via a double sided tape. The tested human body was either in a sitting upright posture or a normal walking condition. Prior to the testing, 15 min resting was required. In this investigation, the testing (sampling) time was up to 30 s. Based on this, the electrical signals acquired from the proposed sensor patch and the previously-reported sensor patch were processed, as aforementioned, to obtain the electrical signals of respirations. For the respiration detection based on the commercial RET (BIOPAC Systems, Inc., SS5LB), a band-type device, was tightly fasten around the chest wall of the human body following the guidance of manufacturer’s instructions. The operation conditions were same as the sensor patch-based detection. The acquired raw electrical signals were subsequently processed with the same scheme previously described so as to obtain the data of respirations for comparison purpose.

For the comparison of the respiratory rate measured by the abovementioned three sensing schemes, further experiments were conducted. In this study, four individuals including two males and two females were tested with the same experimental conditions as previously described except for a longer testing (sampling) time of 3 min. In this study, each test was performed in triplicate (three individual samples) to obtained data for further statistical analysis.

### 2.3. Statistical Analysis

In this study, the data are presented as the mean ± the standard deviation from the triplicate test. Paired *t*-test, a statistical method calculating the difference between a paired measurement, with a statistical significant level of 0.05 was used to compare the data of respiratory rate obtained from the proposed sensor patch, and the commercial RET.

## 3. Results and Discussion

**Figure 3:**
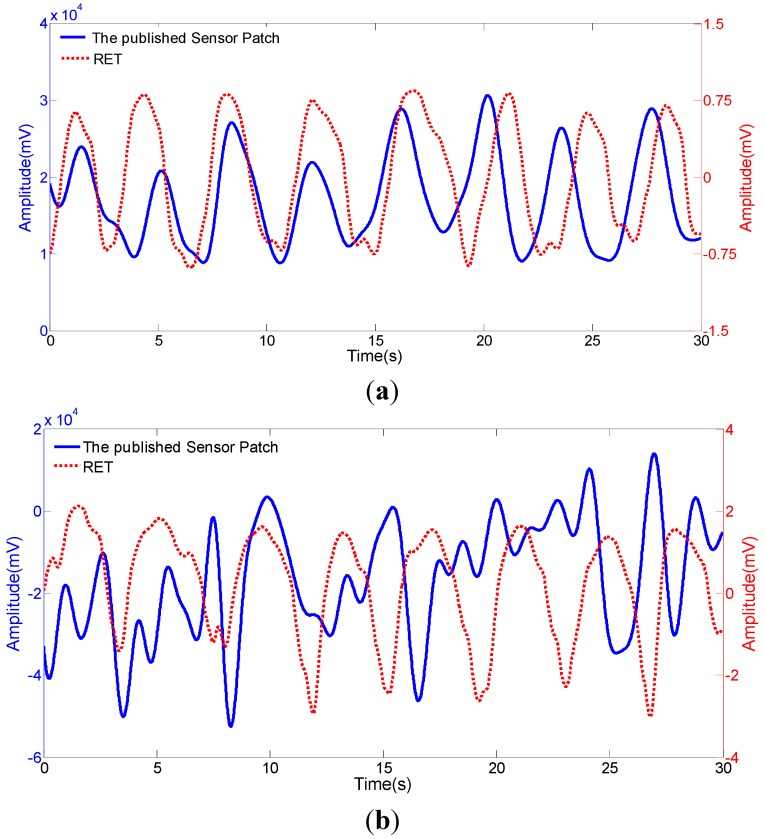
Comparison of the electrical signals acquired from the previously-reported sensor patch, and a commercial respiratory effort transducer under (RET) (**a**) static (sitting), and (**b**) dynamic (walking) conditions.

In our previous study, we have proposed a simple, small-size, light-weight, and user-friendly PVDF polymer-based sensor patch for respiration monitoring [17] as described in the introduction section. In the design, the sensor patch was composed of three layers including a PDMS cover module layer, a PVDF sensing film layer, and a Mylar layer (a hard polyester film). In order to enhance the signal amplitude, the PVDF layer was bonded with the Mylar layer in a curved structure and it can be freely movable. In that system, the PVDF sensing layer can be modeled as a mass-spring mechanical system without damping effect. In general, an undamped mechanical system normally has the characteristic response of large overshoot and long settling time. Hence, the mechanical amplitude of the PVDF layer could be enhanced by large overshoot when the stimulated oscillation was in a regularly repeated manner. Higher mechanical deformation could in turn generate higher electrical signals, as shown in our previous work [17]. It was reported that the electrical signals were around 151% of that based on the sensor with flat PVDF structure under static respiration measurement [17]. However, response with long settling time generated disturbed harmonic oscillation when the stimulated oscillation frequency and the natural frequency of the PVDF layer were not the same. In this situation, the measurement under static conditions (e.g., sitting or sleeping) was still acceptable because the stimulated oscillation frequency was fixed, as shown in Figure 3a. The disturbed harmonic oscillation could be filtered out by the signal conditioning post-process. When the measurement was under dynamic conditions (e.g., walking), conversely, the mechanical response was highly influenced by such disturbed harmonic oscillation with the combination of various frequencies. The generated electrical signals may not represent the actual respiration motions, as shown in Figure 3b. Therefore, the design of the previously-reported sensor patch might not be suitable for the respiration detection particularly under dynamic condition due to noise problem.

**Figure 4:**
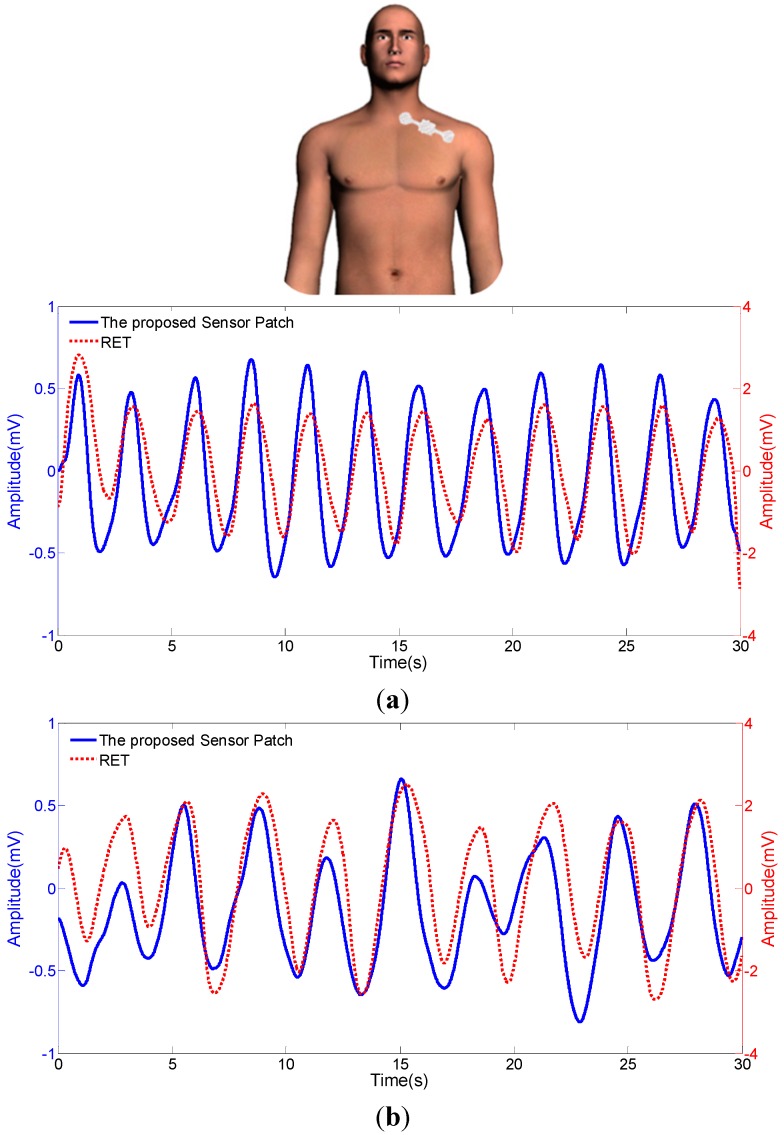
Comparison of the electrical signals acquired from the proposed sensor patch, and a commercial respiratory effort transducer (RET) under (**a**) static (sitting), and (**b**) dynamic (walking) conditions.

In order to tackle the abovementioned technical problem, the proposed PVDF-based sensor patch was developed for the respiration measurement under dynamic walking conditions. In the design, a thin PVDF sensing layer was entirely encapsulated by two elastic PDMS cover layers in order to constraint its free movements which is different from the design of the previously-reported sensor patch [17]. Based on the structure design, the PVDF sensing layer can be modeled as a mass-spring-damper mechanical system. The phenomenon of large overshoot and long settling time normally occurred in an undamped system could be, to great extent, down-regulated. Since the overshoot was reduced, the mechanical amplitude of PVDF sensing layer was accordingly reduced. Therefore, the amplitude of electrical signal output might be compromised. Nevertheless, the reduction of settling time could eliminate the disturbed harmonic oscillation, which could enable the proposed sensor patch to significantly prevent dynamic noises. In order to justify this hypothesis, experimental investigations were carried out. Figure 4a,b revealed the respiration signals generated by the proposed sensor patch under static and dynamic conditions, respectively. It can be clearly found that the respiration signals generated by the proposed sensor patch were in concordance with the commercial RET under the two operating conditions explored. This could suggest that the proposed sensor patch was able to faithfully respond to the physiological movements of respirations under both conditions. For comparison of the respiratory rates measured by the proposed sensor patch and the commercially-available device (commercial respiratory effort transducer (RET)), further experiments were conducted. In this evaluation, four individuals including two males and two females were tested with the same experimental conditions. Table 1 disclosed the measurement results obtained by the proposed sensor patch and a commercial RET under static and dynamic conditions. It was found that there was no statistical difference (*p* > 0.05) between both measurement techniques, indicating the proposed sensor patch holds great promise to faithfully detect respiration signals both in static and dynamic conditions. Subsequent large-scale clinical study might be required to further confirm its accuracy and clinical utility. Based on above experimental evaluations, overall, it can be concluded that, the PVDF-based sensor patches with two different designs (*i.e.*, the previously-reported and the proposed PVDF-based sensor patch) could be selected for the respiratory measurements under different implementing conditions. For the applications situating in static conditions (e.g., sleeping or sitting), for example, the previously-reported sensor patch is suitable because it could generate higher electrical signals for improving the signal-to-noise ratio. On the other hand, the sensor patch proposed in this study could be the ideal choice for the detection of respiration under dynamic conditions (e.g., walking) because of its capability to prevent the generation of dynamic noises. As a whole, this study has presented a simple and portable PVDF-based sensor patch that is capable of detecting respiration signals under static sitting and dynamic walking conditions. These technical features make it an ideal sensing device for the monitoring of respiration in patient home care. In terms of future perspectives, a wireless signal transmission mechanism can be integrated in the sensor patch. By then, the signals generated can be transmitted to a smartphone or other hand-held devices, in which a built-in software for signal processing can be used to analyze the relevant signals.

**Table 1:** Comparison of the respiration rates measured by the proposed sensor patch, and commercial respiratory effort transducer (RET) under static (sitting) and dynamic (walking) conditions; unit: times·min^−1^.

Sex	Age	Static Condition		Dynamic Condition	
		Sensor Patch	RET	Sensor Patch	RET
M	23	7.78 ± 0.38	7.78 ± 0.38	9.34 ± 0	9.34 ± 0
F	23	11.33 ± 0.67	11.33 ± 0.67	12.44 ± 0.77	12.44 ± 0.77
M	24	15.11 ± 0.38	15.11 ± 0.39	18.22 ± 0.38	18.22 ± 0.38
F	24	9.11 ± 0.39	9.11 ± 0.39	10.22 ± 0.38	10.22 ± 0.38
		*p* > 0.05		*p* > 0.05	

## 4. Conclusions

In order to carry out the respiration monitoring in patient home care, a simple, and portable sensing device capable of detecting respiration signals in daily dynamic conditions (e.g., walking) is required. To address this issue, a PVDF-based sensor patch was proposed. In the structure design, the thin PVDF sensing film was completely encapsulated within the elastic PDMS material to form a mass-spring-damper mechanical system. Borrowing from the feature of low settling time in such mechanical system, the phenomenon of disturbed harmonic oscillation occurred in a dynamic condition could be reduced, allowing the sensor patch to be capable of preventing dynamic noises. Experimental evaluations demonstrated the proposed sensor patch can detect respiration signals both in a static (e.g., sitting) or dynamic (e.g., walking) condition. The feasibility of using the proposed sensor patch to carry out respiratory rate measurements was confirmed, in which the results of such measurements have no statistic difference with those based on a commercial respiratory effort transducer under static and dynamic conditions. The subsequent large-scale clinical study might be required to further confirm its accuracy and clinical utility. As a whole, this proof-of-concept study has designed a structure of PVDF-based sensor patch which was capable of monitoring the respirations in a dynamic walking condition with high fidelity. In terms of real application in patient home care, the relevant electric circuit (e.g., an amplifier circuit), electric components (e.g., a microcontroller and a RF transmitter), or a coin battery can be integrated in the proposed sensor patch. Through a wireless signal transmission mechanism, the signals generated can be transmitted to a smartphone or other light weight hand-held devices, in which a built-in software for signal processing can be used to analyze the relevant signals.
